# Alpha-Mangostin Activates MOAP-1 Tumor Suppressor and Mitochondrial Signaling in MCF-7 Human Breast Cancer Cells

**DOI:** 10.1155/2022/7548191

**Published:** 2022-01-17

**Authors:** Samson Eugin Simon, Hui Sin Lim, Fairen Angelin Jayakumar, Ee Wern Tan, Kuan Onn Tan

**Affiliations:** Cancer Biology Group, Department of Biological Sciences, School of Medical and Life Sciences, Sunway University, No. 5 Jalan Universiti, Subang Jaya 47500, Selangor Darul Ehsan, Malaysia

## Abstract

*α*-Mangostin, one of the major constituents of *Garcinia mangostana*, has been reported to possess several biological activities, including antioxidant, anti-inflammatory, antibacterial, and cytotoxic activities associated with the inhibition of cell proliferation and activation of apoptosis. However, the cellular signaling pathway mediated by *α*-mangostin has not been firmly established. To investigate the cellular activities of *α*-mangostin, human cancer cells, MCF-7 and MCF-7-CR cells, were treated with *α*-mangostin to measure the cellular responses, including cytotoxicity, protein-protein interaction, and protein expression. Cancer cells stably expressed Myc-BCL-XL and HA-MOAP-1 were also included in the studies to delineate the cell signaling events mediated by *α*-mangostin. Our results showed that the apoptosis signaling mediated by *α*-mangostin involves the upregulation of endogenous MOAP-1, which interacts with *α*-mangostin activated BAX (act-BAX) while downregulating the expression of BCL-XL. Moreover, *α*-mangostin was found to induce BAX oligomerization, the release of mitochondrial cytochrome C, and activation of caspase in MCF-7 cells. In overexpression studies, MCF-7 cells and spheroids stably expressed HA-MOAP-1 and Myc-BCL-XL exhibited differential chemosensitivity toward *α*-mangostin in which the stable clones expressing HA-MOAP-1 and MYC-BCL-XL were chemosensitive and chemoresistant to the apoptosis signaling events mediated by *α*-mangostin, respectively, when compared to untreated cells. Together, the data suggest that the cytotoxicity of *α*-mangostin involves the activation of MOAP-1 tumor suppressor and its interaction with act-BAX, leading to mitochondria dysfunction and cell death.

## 1. Introduction

Natural products derived from *G. mangostana* L. *(Clusiaceae)* have been used traditionally for wound healing or as antimicrobial and antiparasitic agents to treat dysentery or skin infection in many parts of Asia, including Malaysia, Thailand, and China [1–3]. The anti-inflammatory property of *G.mangostana* has been exploited for the treatments of eczema and psoriasis [4–6]. Phytochemical studies of *G.mangostana* have identified xanthones as the major constituents, particularly *α*-mangostin [7–10].

In addition, natural products derived from *G. mangostana* are known to exhibit cytotoxic effects on cancer cells [4, 11–14]. However, the mechanisms of action of these natural products have not been firmly established. In fact, *α*-mangostin isolated from *G. mangostana* has been reported to mediate ROS-associated cytotoxicity [12, 15], apoptosis, anti-inflammatory, and antimicrobial activities [12, 16]. Moreover, *α*-mangostin has been shown to inhibit the expression of MMP2 and MMP9 and to promote the release of endonuclease-G from mitochondria through caspase-independent apoptosis [12, 14, 17].

Conventional chemotherapies rely on small molecule chemotherapy drugs to induce cytotoxicity in fast-dividing cancer cells, including small molecules derived from natural resources such as xanthones isolated from *G. mangostana* that were reported to mediate cytotoxic effects on cancer cells [18, 19]. However, cancer cells are known to develop resistance to chemotherapy, rendering chemotherapy drugs ineffective, and higher doses of chemotherapy drugs may be required to achieve the therapeutic outcome [20–23]. In addition, cancer treatments that utilize high doses of chemotherapy drugs are associated with cellular cytotoxicity, including the weakening of the immune system that gives rise to opportunistic infections [24–26]. Therefore, high potency anticancer drugs that exhibit high specificity and low cytotoxic effects are likely to be efficacious for cancer treatment by targeting specific features of cancer cells, including the mitochondria which are the prime source of ATP production that is required to sustain the growth of cancer cells [27, 28].

One of the important mitochondrial signaling molecules is BAX, which is a proapoptotic member of the Bcl-2 family, and it is activated by a receptor-mediated signaling mechanism that involves MOAP-1 and RASSF1A through TNF and TRAIL receptors mediated apoptotic signaling mechanism [29–33]. MOAP-1 takes part in the apoptotic signaling by interacting with BAX through its BH3-like domain, leading to BAX activation and oligomerization at the mitochondria [23, 34–37]. Oligomerization of BAX induces depolarization of mitochondria membrane potential and release of mitochondrial cytochrome C that ramps up the activation of apoptosome and caspases [30, 33, 38–44].

To evaluate the cytotoxicity of *α*-mangostin and to determine its potential interaction with molecules associated with mitochondria, both endogenous and overexpressed mitochondria-associated proteins, including MOAP-1 and BCL-XL, were included in this investigation. Overexpression of the exogenous proteins in the cancer cells offers added advantages in which the cellular activity of the overexpressed protein in the *α*-mangostin treated cells can be studied, evaluated, and compared to other proteins, including negative control, that are similarly overexpressed under the same experimental paradigms. Our results showed that *α*-mangostin activates downstream apoptosis signaling events, leading to mitochondria dysfunction and cell death.

## 2. Methods

### 2.1. Reagents and Antibodies

*α*-Mangostin (99% purity, prepared in DMSO), AlamarBlue^®^ cell viability reagents, DAPI, Annexin-V apoptosis reagent, antimouse secondary antibody conjugated to Horseradish Peroxidase, and Protein A agarose were purchased from Thermofisher (Waltham, MA, USA). Z-VAD-fmk, a pan-caspase inhibitor, was obtained from MedChem Express (Monmouth Junction, NJ, USA). Antibodies against MOAP-1 (A11, Sc-271338), BCL-XL (H-5, sc-8392), BAX (6A7, sc-23959, sc-7480), BAX (N20, sc-493), Cytochrome C (A8, sc-13156), HSP60 (F9, sc-376261), and *β*-ACTIN (C4, sc-47778) were purchased from Santa Cruz Biotechnology (USA). Polyethyleneimine (Linear, MW 25000) was obtained from PolyScience (Niles, IL, USA). Caspase assay using pGLOSensor™-30F DEVD plasmid and ONE-Glo™ luciferase reagent was purchased from Promega (Madison, WI, USA). BMH crosslinking reagent was purchased from Tokyo Chemical Industry (Tokyo, Japan).

### 2.2. Cell Culture

The human breast epithelial cancer cell lines, MCF-7 (ATCC® HTB-22™) and MCF-7-CR [45], which is a cisplatin-resistant clone of the MCF-7 cells and HaCaT cells (CLS Cell Lines Service, Germany, Cat: 300493) were maintained in Dulbecco's Modified Eagle Medium (DMEM) supplemented with 10% fetal bovine serum (FBS; Gibco), 2 mM glutamine, 100 U/ml penicillin, and 100 *µ*g/ml streptomycin (Sigma), at 37°C in a humidified atmosphere with 5% CO_2_.

### 2.3. Cell Viability Assay and Selectivity Index

MCF-7, MCF-7-CR, and HaCaT cells were seeded in a 96-well plate at a density of 1.5 × 10^4^ cells/well and allowed to grow for 48 h. The cells were then treated with various concentrations of *α*-mangostin. Cell viability was determined using Alamar Blue or MTT reagent, and the percent cell viability was normalized against untreated cells. The dose-dependent inhibition concentrations (IC_50_) of *α*-mangostin were determined in MCF-7 and MCF-7-CR cells using Graph-Pad Prism. To determine the selectivity index of *α*-mangostin, the IC50 value of HaCaT cells was divided by the respective IC50 value of MCF-7 and MCF-7-CR cells.

### 2.4. Stable Clone Selection

MCF-7 cells were seeded in a 60 mm plate and transfected for 6 h with 5 *µ*g of pcDNA-HA-MOAP-1, Myc-BCL-XL, or the vector. After transfection, cells were treated with 800 *µ*g/ml of geneticin (G418) sulfate (Santa Cruz, USA) for three weeks. Media containing 800 *µ*g/ml of G418 was changed once every three days. The selected stable clones were isolated using cloning rings and expanded for experiments while cultured in media containing 400 *µ*g/ml of G418.

### 2.5. Spheroid Cell Depletion

MCF-7 spheroid cells were generated by dropping 30 *µ*l of media containing 1 × 10^3^ of MCF-7 cells on UV treated sterile glass coverslip and inverted over in each well of 24 well plates containing sterile 1x PBS. After incubating for 48 h, 0.2% agarose gel at a temperature less than 50°C was prepared in DMEM containing 40% FBS and 100 *µ*l of the agarose gel mixture was transferred into a 24 well plate without air bubble and allowed to solidify. Hanging cells were then flushed with 100 *µ*l of 10% FBS complete media into wells containing the agarose gel mixture. The depletion of the spheroid mass was obtained by treating the spheroid cells with *α*-mangostin and the cell viability of the spheroids was measured using Alamar Blue periodically from 0–5 days. The spheroid cell depletion was also monitored periodically for up to 5 days with the images captured under a microscope.

### 2.6. Flow Cytometry

Apoptosis of cancer cells was detected using Annexin-V APC and PI, according to the manufacturer's instruction. In brief, 4 × 10^5^ of MCF-7 cells were seeded in 6-well plates at 80% confluency for 48 h followed by treatment with *α*-mangostin (10, 20, and 30 *µ*M) overnight. The cells were subsequently stained with Annexin V and PI in the dark for 15 min followed by flow cytometry analysis (FACS Calibur, BD Biosciences).

### 2.7. Nuclear Condensation Assay

MCF-7 cells at a density of 4 × 10^5^ cells/well were seeded on a sterile glass coverslip in 6-well plates for 48 h and treated with *α*-mangostin (10, 20, and 30 *µ*M). The cells were subsequently fixed using ice-cold methanol and stained with DAPI. Condensed nuclei were identified using a fluorescence microscope by counting the number of DAPI stained cells from five randomly selected fields under the fluorescence microscope (Nikon).

### 2.8. Western Blotting and Coimmunoprecipitation

MCF-7 cells were seeded equally in a 60 mm plate at a density of 1.4 × 10^6^ cells/well. After 48 h, the cells were treated with *α*-mangostin for 12 h. The cells were collected using a cell scraper and washed with 1x PBS. RIPA buffer (50 mM Tris-HCl, pH 7.5, 150 mM NaCl, 1 mM EDTA, 0.25% Na-deoxycholate, 1% NP-40) containing protease inhibitors was added to the cells followed by incubation on ice for 15 min. The cell pellets were vortexed to lyse the cells, and clear cell lysates were collected from the supernatants by centrifuging the cell lysates at 15000 xg for 10 min. Equal amounts of the cell lysates, as determined by protein concentration, were separated on 12.5% SDS-PAGE followed by Western blot analysis with antibodies to detect MOAP-1, BCl-XL, BAX, or *ß*-ACTIN. Primary antibodies (1 : 1000) were probed overnight at 4°C, and a secondary antibody conjugated to HRP (1 : 5000) was probed for 1 h at room temperature. For coimmunoprecipitation, MCF-7 cells were seeded equally in a 100 mm plate at a density of 2.5 × 10^6^ cells *per* plate and treated as mentioned earlier. Cell lysates were prepared with CHAPS buffer (20 mM Tris-HCl, pH 7.5, 150 mM NaCl, 0.2% Nonidet P-40, 1 mM EDTA, protease inhibitors), and polyclonal anti-BAX antibody (N20) was added to the cell lysate for overnight incubation at 4°C. Protein A agarose beads were subsequently added to the cell lysates followed by 4 h of incubation at 4°C. The immunoprecipitates were analyzed through Western blot with the indicated antibodies. Signals were visualized using ECL reagent (Pierce) and detected using a Luminescent Image Analyzer LAS-4000 mini.

### 2.9. Mitochondrial Cytochrome C Release Assay

MCF-7 cells were seeded in a 60 mm plate at a density of 4 × 10^6^ cells/plate. After 48 h, the cells were treated with *α*-mangostin (10, 20, and 30 *µ*M) for 12 h. The cells were subsequently harvested and suspended in lysis buffer A (10 mM HEPES, pH 7.9, 10 mM KCl, 0.1 mM EDTA, 1 mM dithiothreitol) containing protease inhibitors followed by 15 min incubation on ice and vortexed. Using 25^5/8^ gauge needle, the cell suspension was expulsed through the needle 25 to 30 times and centrifuged at 1000 ×g for 10 min to remove cell debris and nucleus. The supernatants containing cytosolic fractions were subsequently centrifuged for 10 min at 3000 xg to pellet the mitochondria. The pellets were washed once with buffer A containing protease inhibitors and suspended in RIPA buffer (50 mM Tris at pH 7.5, 150 mM NaCl, 1 mM EDTA, 0.25% Na-deoxycholate, 1% NP-40) containing protease inhibitors. The lysate was collected from the supernatant by centrifuging at 15000 xg for 10 min followed by Western blot analysis using the indicated antibodies, including anti-Cytochrome C and HSP60 antibodies. The signals were visualized using ECL reagent (Pierce) and detected using a Luminescent Image Analyzer LAS-4000 mini.

### 2.10. Transfection and Caspase Assay

All transfection assays were performed using polyethyleneimine (PEI, Polyscience, Inc.). pGLO sensor-30F DEVD plasmid was used for caspase assay, which was performed using the Luciferase One-Glo assay kit (Promega, USA) according to the manufacturer's protocol. MCF-7 cells or MCF-7 stable clone cells were seeded in 24-well plates for caspase assay. At 70% confluency, 0.5 µg of pGLO sensor-30F DEVD plasmid was mixed with PEI (1 : 3) and incubated in serum-free media before adding the mixture to the MCF-7 cells for transfection. After 4 h of transfection, fresh media containing 10% FBS was added and incubated in a 37°C incubator for 24 h followed by treatment with *α*-mangostin for 12 h. For cells treated with Z-VAD-fmk caspase inhibitor, the inhibitor was added 1 h before the *α*-mangostin treatment. The cells were harvested and One-Glo luciferase reagent was added to the cell lysate, and fluorescent readings were obtained through the multiplate reader and normalized with the readings obtained from control cells.

### 2.11. BMH Chemical Cross-Linking

MCF-7 cells were seeded equally in a 100 mm plate at a density of 2.5 × 10^6^ cells. At 80% confluency, the MCF-7 cells were treated for 8 h with 30 uM *α*-mangostin. The cells were harvested and lysed in CHAPS buffer (20 mM Tris, pH 7.5, 150 mM NaCl, 0.2% Nonidet P-40, 1 mM EDTA, protease inhibitors). Protein concentration was quantified using BCA reagent according to the manufacturer's protocol, and 0.5 mg of protein was mixed with 0.5 ml 2X PBS along with100 *μ*M BMH crosslinking reagent for 45 min. The reaction was subsequently quenched using 0.5 mM DTT and incubated for 15 min. Western blot was performed as mentioned previously, with the indicated antibody.

### 2.12. Statistical Analysis

The results are presented as mean ± standard error (S.E.) from three independent experiments. Data were analyzed using the GraphPad Prism 8.0 or Excel Spreadsheet. Student's *t*-test or ANOVA was performed to determine whether the observed differences in the data were statistically significant as represented by *p*-value, *p* < 0.05 or *p* < 0.001.

## 3. Results

**Figure 1:**
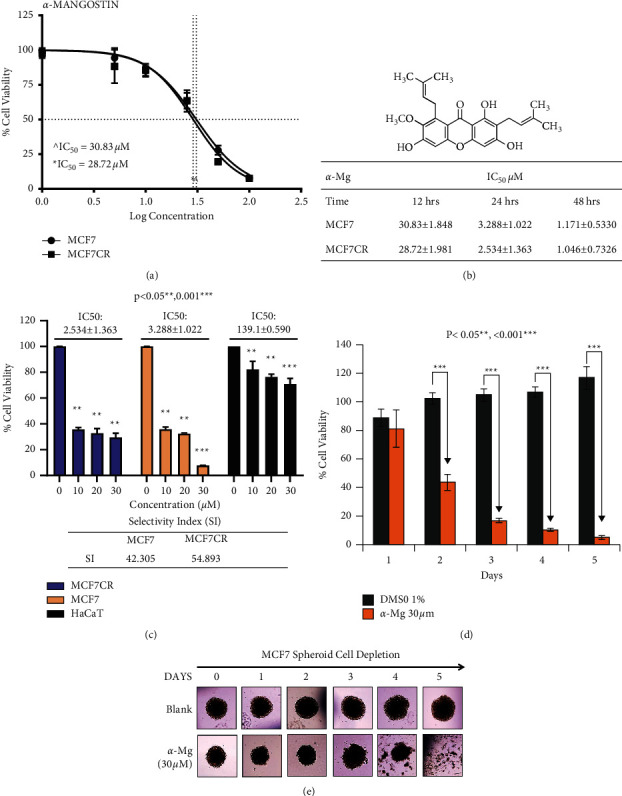
Cytotoxic effects of *α*-mangostin on cancer cell lines and spheroid cells. (a, b) Cytotoxicity of *α*-mangostin on human cell lines. The cell lines were treated with different concentrations of *α*-mangostin for 12 h (a) or extended up to 48 h (b) and the cell viability of the cells was measured to determine the IC50 values. (c) Selectivity index (SI) of *α*-mangostin. The cells were treated with the indicated concentration of *α*-mangostin, and the IC50 values of the treated cells were obtained. SI value of *α*-mangostin was determined in MCF-7 and MCF-7-CR as described in Methods section. (d) Time-dependent loss of cell viability of MCF-7 spheroid cells treated with *α*-mangostin. The cell viability of the spheroid cells was measured after treatment with *α*-mangostin at the indicated time. For control, the cells were treated with 1% DMSO (vol/vol) in the cell culture media, and the results from the control were used to normalize the data. (e) Morphology of *α*-mangostin-treated MCF-7 spheroid cells.

To characterize potential novel cell signaling pathways mediated by *α*-mangostin in cancer cells, MCF-7 and MCF-7-CR cells were treated with different concentrations of *α*-mangostin, and the IC50 and selectivity index values based on the cell viability of *α*-mangostin-treated cells were determined (Figures 1(a)–1(c)). The cytotoxicity of *α*-mangostin on MCF-7 spheroid cells was also examined (Figures 1(d)-1(e)). In these models, *α*-mangostin induced both time- and dose-dependent loss of cell viability on the human cancer cells.

**Figure 2:**
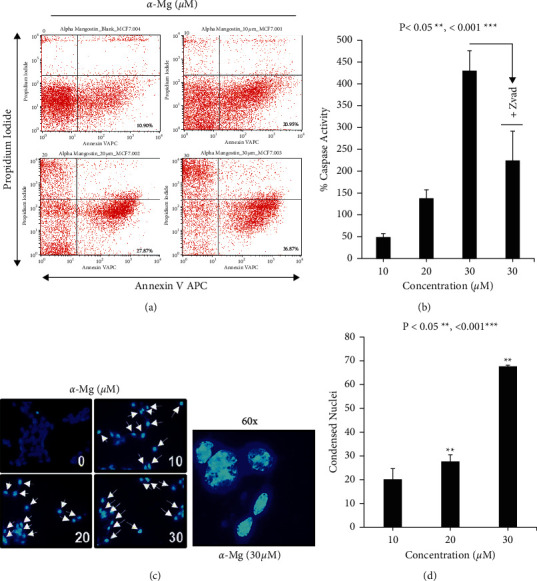
*α*-Mangostin promotes apoptosis in MCF-7 cells. (a) *α*-Mangostin induced apoptotic cell death in MCF-7 cells with Annexin-V positive staining. MCF-7 cells were treated with different concentrations of *α*-mangostin followed by staining with Annexin-V and propidium iodide before flow cytometry analysis. (b) *α*-Mangostin induced apoptotic cell death in MCF-7 cells through the activation of caspase. The caspase activity of *α*-Mangostin-treated cells was measured using pGLO sensor −30F DEVD. (c) *α*-Mangostin induced nuclei condensation in MCF-7 cells. MCF-7 cells were treated with different concentrations of *α*-mangostin followed by DAPI staining and imaging using fluorescence microscopy. (d) Quantitative measurement of condensed nuclei in MCF-7 cells is described in (c).

To determine whether the cytotoxic effect of *α*-mangostin involves necrosis or apoptosis, MCF-7 cells were treated with *α*-mangostin for 18 h, and the cells were stained with Annexin-V and propidium iodide followed by flow cytometry analysis. As shown in Figures 2(a)–2(d)), *α*-mangostin induced dose-dependent apoptotic cell death in MCF-7 cells, resulting in positive staining with Annexin-V, caspase activation, and nuclei condensation, indicating *α*-mangostin possesses high potency in inducing apoptotic cell death in MCF-7 cells.

**Figure 3:**
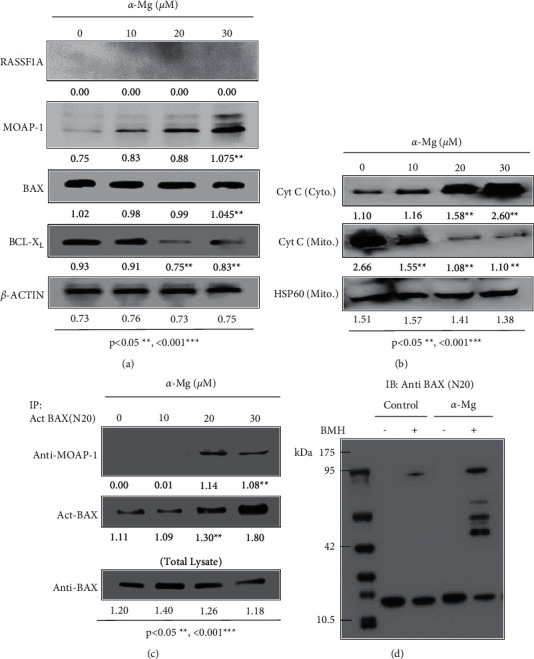
*α*-Mangostin induces the upregulation of endogenous MOAP-1 and promotes the interaction of MOAP-1 with activated BAX. (a) *α*-Mangostin induced the upregulation and the downregulation of endogenous MOAP-1 and BCL-XL, respectively. MCF-7 cells were treated with different concentrations of *α*-mangostin, and the cell lysates were prepared for immunoblot analysis using antibodies specific for each of the indicated proteins. (b) *α*-Mangostin induced the release of mitochondrial Cyt. (C) After treatment, the cytosol and mitochondria fractions were prepared for immunoblot analysis. (c) *α*-Mangostin promoted the interaction of MOAP-1 with activated BAX in MCF-7 cells. After treatment, the cell lysates were prepared for coimmunoprecipitation with N20 antibody specific for activated BAX (Act. BAX) followed by immunoblot analysis of the immunoprecipitants. (d) Oligomerization of activated BAX in MCF-7 cells treated with *α*-mangostin (30 *µ*M). After treatment, the cell lysates were prepared for crosslinking with BMH followed by immunoblot analysis with an anti-BAX antibody. The protein bands were quantified using ImageJ [46].

To identify the mechanism of apoptosis mediated by *α*-mangostin, MCF-7 cells were treated with different concentrations of *α*-mangostin, and the cell lysates were prepared for Western blot analysis with antibodies specific for the proapoptotic and antiapoptotic proteins, including BAX, MOAP-1, and BCL-XL. As shown in Figure 3(a), MCF-7 cells treated with *α*-mangostin showed dose-dependent upregulation and downregulation of endogenous MOAP-1 and BCl-XL proteins, respectively, while the protein level of proapoptotic BAX remained relatively constant under the same treatment condition (Figure 3(a)). In addition, the cellular effects of *α*-mangostin coincided with its activity in mediating the dose-dependent release of mitochondrial Cyt. C in MCF-7 cells treated with *α*-mangostin (Figure 3(b)).

Since BAX was reported to interact with MOAP-1, the level of BAX activation and its association with MOAP-1 were determined using a coimmunoprecipitation assay. As shown in Figure 3(c), MCF-7 cells treated with increasing concentration of *α*-mangostin showed dose-dependent upregulation of activated BAX (act-BAX), which coimmunoprecipitated with MOAP-1 (Figure 3(c)). The activated BAX was shown to coimmunoprecipitate with MOAP-1 when MCF-7 cells were treated with *α*-mangostin (Figures 3(c)), which at these concentrations coincided with the substantial release of Cyt. C from mitochondria (Figure 3(b)), suggesting that *α*-mangostin induces apoptosis signaling by promoting the interaction of act-BAX with MOAP-1, leading to the release of mitochondrial Cytochrome C. Furthermore, *α*-mangostin induced oligomerization of BAX (Figure 3(d)), and the BMH cross-linked BAX oligomers were identified by the anti-BAX antibody.

**Figure 4:**
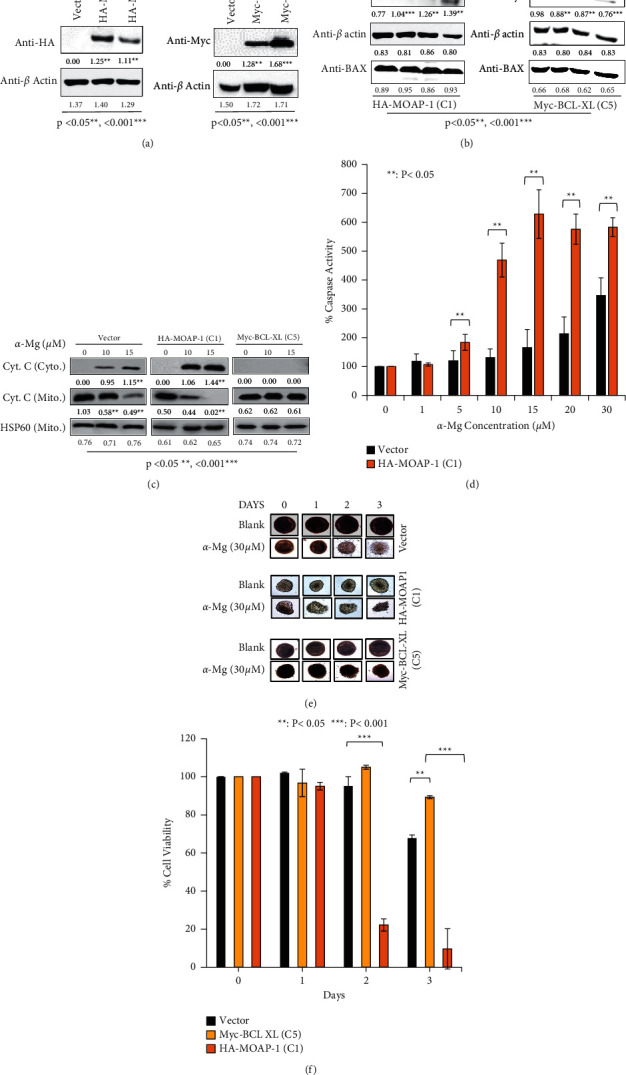
*α*-Mangostin enhances chemosensitization of MCF-7 cells and spheroids stably expressed HA-MOAP-1. (a) Western blot analysis of MCF-7 stable clones stably expressed HA-MOAP-1 or Myc-BCL-XL. The cell lysates of the stable clones were prepared and analyzed on a Western blot using the indicated antibodies. (b) The upregulation of HA-MOAP-1 and downregulation of Myc-BCL-XL proteins in the MCF-7 stable clones treated with *α*-mangostin. The cell lysates of the stable clones were prepared and analyzed with the indicated antibodies. *α*-Mangostin increased dose-dependent release of mitochondrial Cyt. C (c) and activation of caspase (d) of HA-MOAP-1 stable clone. The cytosolic and mitochondria fractions were prepared for immunoblot analysis with the indicated antibodies. The caspase activity of the treated cells was measured using pGLO sensor-30F DEVD. (e) Depletion of MCF-7 spheroid cells stably expressed HA-MOAP-1 by *α*-mangostin. Morphology of the spheroid MCF-7 cells treated with *α*-mangostin. (f) Quantification of the cell viability of the spheroid cells as described in (e). Cell viability of the spheroid cells was quantified at the indicated time after treatment with *α*-mangostin. The protein bands were quantified using ImageJ [46].

To further substantiate the role of MOAP-1 in *α*-mangostin-mediated apoptosis signaling, stable clones of MCF-7 cells overexpressed HA-MOAP-1 or Myc-BCL-XL were generated, and the expressed proteins were detected using Western blot (Figure 4(a)). HA-MOAP-1 and Myc-BCl-XL were dose-dependently upregulated and downregulated, respectively, when the respective stable clones were treated with *α*-mangostin (Figure 4(b)). In addition, the stable clone expressed HA-MOAP-1 showed a high level of chemosensitization toward *α*-mangostin when compared to Myc-BCL-XL or vector stable clone as evidenced by the dose-dependent release of mitochondrial Cyt.C and activation of caspase (Figures 4(c)-4(d)).

The MCF-7 stable clones were further evaluated using 3D spheroid cell model in which the spheroids were treated with *α*-mangostin for 3 days. Spheroid cells expressed HA-MOAP-1 responded the most to the treatment with *α*-mangostin, resulting in time-dependent loss of cell viability in which the spheroid cells lost close to 90% of their cell viability after 3-day of treatment with *α*-mangostin (Figures 4(e)–4(f)). In comparison, the spheroid cells expressed Myc-BCl-XL or vector lost 11% or 32% of cell viability, respectively, under the same treatment (Figures 4(e)-4(f)), suggesting that *α*-mangostin activates MOAP-1-mediated apoptosis signaling, while BCL-XL confers protection and antagonizes the apoptosis signaling mediated by *α*-mangostin in MCF-7 cells.

## 4. Discussion

**Figure 5:**
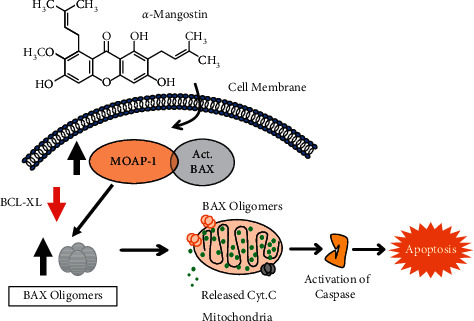
Schematic representation depicting the responses of MOAP-1, BAX, BCL-XL, and mitochondria to *α*-mangostin.

Small molecules (<500 Daltons) that are targeting cell signaling pathways offer an alternative treatment option for cancer. To identify high potency bioactive compounds, bio-assays were used to characterize the cytotoxicity of *α*-mangostin, including its potential interaction with the mitochondria signaling pathway. Our results showed that *α*-mangostin exhibits selective cytotoxicity against MCF-7 and MCF-7 cells with relative high selective index (SI > 40), when compared to HaCaT cells [47, 48]. The selective cytotoxicity of *α*-mangostin was also reported in leukemic cancer cells [49]. The cytotoxicity of *α*-mangostin involved activation of apoptosis signaling mediated by MOAP-1 and the interaction of MOAP-1 with act-BAX, leading to oligomerization of BAX, the release of mitochondrial cytochrome C, and apoptosis (Figure 5). The results are consistent with the reported findings that showed that *α*-mangostin induced apoptosis by activating cell cycle arrest or ASK/*p*38 signaling pathway in oral squamous cell carcinoma and cervical cancer cells, respectively [50, 51]. Taken together our data thus suggest that the apoptosis signaling mediated by *α*-mangostin in human cancer cells that involves the activation of MOAP-1 and BAX is likely to impact on other signaling pathways activated by *α*-mangostin, such as cell cycle arrest, leading to apoptotic cell death. The role of mitochondria in the regulation of cell cycle and cell proliferation has been reported [52].

Interestingly, when MCF-7 cells were treated with *α*-mangostin, the protein level of the BCL-XL decreased dose-dependently. The downregulation of BCL-XL mediated by the activators of apoptosis was previously reported [53, 54] and the mechanisms involved protein degradation mediated by proteasomal pathway and cleavage by proteolytic enzyme. As BCL-XL is known to interact with both proapoptotic BAX and MOAP-1 [36, 55–58], the downregulation of BCL-XL is likely to facilitate the activation of apoptosis signaling mediated by MOAP-1 and BAX, leading to BAX oligomerization and mitochondrial dysfunction. The data were further supported by the evidence obtained from MCF-7 cells and spheroids stably expressed HA-MOAP-1, which showed enhanced chemosensitization mediated by *α*-mangostin. In contrast, the MCF-7 cells and spheroids stably expressed Myc-BCL-XL were resistant to chemosensitization mediated by *α*-mangostin even when the protein level of Myc-BCL-XL was reduced due to protein degradation.

MOAP-1 protein level was shown to be stabilized or upregulated by various apoptosis stimuli with different mechanisms of action, including FTY720, Etoposide, TRAIL, and Thapsigargin, suggesting potential cross-talks and convergence of cell signaling mechanism [59, 60]; however, this study highlights that *α*-mangostin promotes the upregulation of MOAP-1 and interaction of MOAP-1 with act-BAX leading to oligomerization of BAX, down-regulation of BCL-XL, the release of mitochondrial Cyt. C, and activation of caspase. Since both endogenous and overexpressed MOAP-1 proteins were upregulated by *α*-mangostin in *α*-mangostin-treated MCF-7 cells independent of gene promoter, the mechanisms of action of *α*-mangostin may involve post-translational stabilization of MOAP-1 protein.

The proteasome inhibitor, MG132, was reported to stabilize polyubiquitinated MOAP-1 from degradation by proteasome, while UBR5 and APC/C^cdh1^ E3 ubiquitin ligases target MOAP-1 for degradation [59, 61, 62]. Besides, TRIM39 was reported to promote the stabilization of MOAP-1 through negative regulation of APC/C^cdh1^ [61, 63]. The mechanism in which *α*-mangostin mediates upregulation of MOAP-1 warrants further investigation to identify the potential regulation of MOAP-1 protein or its transcript by *α*-mangostin. Also, strategies that lead to enhanced expression and stabilization of MOAP-1 protein in cancer cells are likely to confer tangible benefits for therapeutic intervention in cancer. Future investigations are required to further examine the potential impact of *α*-mangostin for cancer treatment.

## 5. Conclusions

The cytotoxic activity of *α*-mangostin in human cancer cells involves the upregulation of MOAP-1 tumor suppressor and activated BAX as well as the interaction of the upregulated MOAP-1 with act-BAX, leading to apoptosis signaling, including BAX oligomerization, downregulation of BCL-XL, the release of mitochondrial Cyt.C, and caspase activation. Cancer cells overexpressing MOAP-1 exhibit enhanced sensitivity, while cancer cells overexpressing BCL-XL are resistant to *α*-mangostin-mediated apoptosis, suggesting that *α*-mangostin induces apoptosis through mitochondria signaling pathway involving the activation of both MOAP-1 and act-BAX while downregulating the expression of BCL-XL.

## Data Availability

Data are available upon request by contacting the corresponding author.

## Abbreviations

KDa: KiloDalton
FBS: Fetal bovine serum
CHAPS: 3-[(3-Cholamidopropyl) dimethylammonio]-1-propanesulfonate
EDTA: Ethylenediaminetetraacetic Acid
SDS-PAGE: Sodium dodecyl sulfate polyacrylamide gel electrophoresis
APC: Allophycocyanin
MTT: 3-(4,5-Dimethylthiazol-2-yl)-2,5-diphenyltetrazolium bromide
PI: Propidium iodide
RIPA: Radio-immunoprecipitation assay
ECL: Enhanced chemiluminescence
HEPES: (4-(2-hydroxyethyl)-1-piperazineethanesulfonic acid
PEI: Polyethyleneimine
BCA: Bicinchoninic acid assay
DTT: Dithiothreitol
FTY 720: Fingolimod
*µ*M: Micromolar
MG132: N-[(phenylmethoxy)carbonyl]-L-leucyl-N-[(1R)-1-formyl-3-methylbutyl]-L-leucinamide
TNF: Tumor necrosis factor
TRAIL: TNF-related apoptosis-inducing ligand
MOAP-1: Modulator of apoptosis 1
PBS: Phosphate-buffered saline
DAPI: 4′,6-Diamidino-2-phenylindole
BMH: Bismaleimidohexane
DMSO: Dimethyl sulfoxide
DMEM: Dulbecco's modified eagle medium
TLC: Thin layer chromatography
HPLC: High-performance liquid chromatography
IB: Immunoblot
Cyt.C: Cytochrome C
IP: Immunoprecipitation.
